# Solitary Tibial Osteolytic Lesion

**DOI:** 10.1155/2009/352085

**Published:** 2009-06-16

**Authors:** Emilios E. Pakos, Dimitrios N. Gartzonikas, Pericles G. Tsekeris, Theodore A. Xenakis

**Affiliations:** ^1^Department of Orthopaedic Surgery, School of Medicine, University Hospital of Ioannina, University of Ioannina, 45500 Ioannina, Greece; ^2^Department of Radiation Therapy, School of Medicine, University Hospital of Ioannina, University of Ioannina, 45500 Ioannina, Greece

## Abstract

We report an unusual case of solitary osteolytic tibial metastasis from a primary endometrial cancer in a 62-year-old woman. The primary cancer was treated with total abdominal hysterectomy and bilateral salpingo-oophorectomy combined with postoperative external beam radiotherapy, while the tibial metastasis was treated with an above knee amputation. The rarity of the case lies on the fact that metastases distally to the elbow and knee are uncommon and endometrial cancer rarely gives distal bone metastases and particularly solitary to the extremities.

## 1. Introduction

Metastatic cancer to the skeleton is the most common malignant bone tumors. Despite the improved efficacy of cancer treatment that resulted to prolonged survival rates during the last years, the prevalence of bone metastases was further increased. Consequently, orthopaedic surgeons are more likely involved in the diagnosis and treatment of this form of bone tumor and particularly the solitary ones. In a large autopsy series of 1000 metastatic cases, bone metastases were found in 27% [1]. The most common localization is the spine [1], while metastases distal to the elbow and knee are rare [2]. Tibia is the most common site when the metastasis is distally to the knee joint [2]. Primary neoplasms that metastasise to the skeleton are prostate, breast, lung, and kidney cancer [1].

Solitary skeletal metastases are the most challenging to validate. A true isolated bone metastasis seldom occurs, with an incidence of only 2-3% of all skeletal spread situations [3]. Their distribution to the skeleton is similar to multiple lesions with the vertebrae been the commonest localization. Solitary bone metastases might be osteolytic, osteoblastic, or mixed. The majority of them are asymptomatic. If not, the main presenting complaint is persistence of bone pain usually unrelieved with nonsteroidal anti-inflammatory drugs. A pathologic fracture might be the first symptom. The differential diagnosis of a solitary metastasis from a primary benign or malignant bone tumor remains a diagnostic challenge.

Endometrial adenocarcinoma usually metastasises to lymph nodes, lung, and liver [4]. Bone metastases from endometrial cancer are very rare with a reported frequency of 0–8% [4–6]. When seen, they are usually localized to the axial skeleton, usually seen together with abdominopelvic recurrences and/or other organ multiple metastases. Metastasis to the extremities is extremely rare [7]. In the present study we report a case of a solitary osteolytic lesion to the tibia in a woman with a history of endometrial cancer.

## 2. Case Presentation

In August 2006, a 62-year-old woman was admitted to the Orthopaedic Department of the University Hospital of Ioannina, Greece, with pain to the distal third of the right tibia and to the right ankle. She reported a 2-month history of pain which was initially intermittent, gradually progressed in intensity and became constant, with no history of previous trauma. The pain was not relieved with nonsteroidal anti-inflammatory drugs. Weight bearing activities exacerbated the symptoms. The patient had a history of total abdominal hysterectomy and bilateral salpingo-oophorectomy in January 2006 due to a stage II endometrial cancer. The histological evaluation showed grade III endometrial endometrioid adenocarcinoma with areas of undifferentiated carcinoma. The latter component had morphological characteristics of a small-cell carcinoma, where a proliferation of small-size epithelial cells growing in solid sheets was observed. Consequently, at the 45th postoperative day the patient underwent external beam radiotherapy with a linear accelerator (6 MV) and a total radiation dose of 45 Gy and intracavitary brachytherapy of 25 Gy with a high-dose-rate Co-60 unit. All clinical and imaging examinations after the end of radiotherapy were negative for the presence of local or systemic disease.

**Figure 1:**
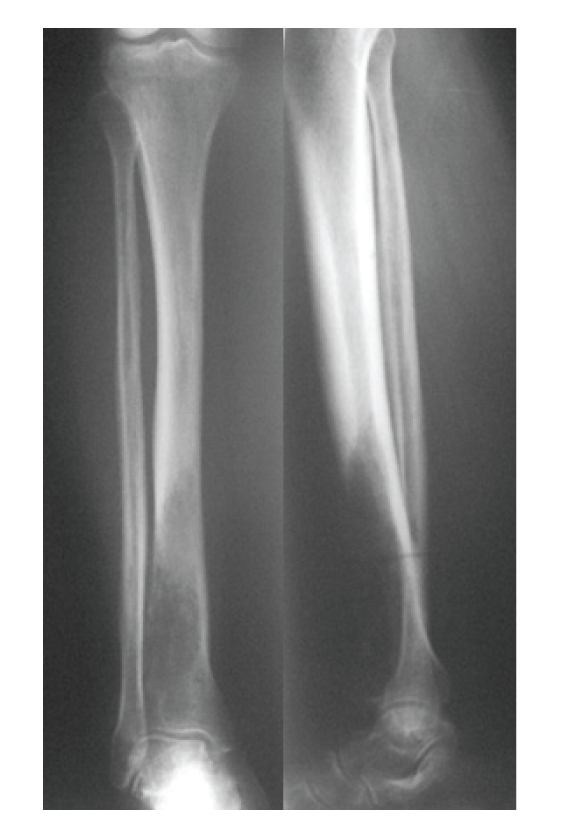
Radiograph showing an osteolytic lesion to the distal third of the tibia with cortical destruction.

**Figure 2:**
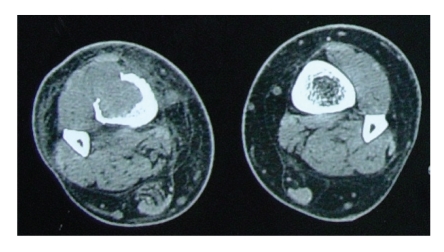
CT-scan of the tibia showing an intramedullar mass which destructs the cortical bone and spreads into the adjacent soft tissues.

On physical examination swelling of the distal third of the right tibia with a palpable soft-tissue mass to the frontal plane was observed. Apart from a slight discolouration of the overlying skin, no other pathological findings were noticed. The laboratory evaluation (complete blood count with differential erythrocyte sedimentation rate, chemistry group, serum protein electrophoresis, and urinalysis) was normal except a slight elevation of C-reactive protein and erythrocyte sedimentation rate. The alkaline phosphate level was normal. Plain radiographs revealed an osteolytic lesion to the distal third of the tibia with cortical destruction (Figure 1). The CT-scan of the tibia revealed an intramedullar mass which destructs the cortical bone and spreads into the adjacent soft tissues (Figure 2). The technetium bone scan did not reveal any occult malignancies. The CT-scan of chest and abdomen had no pathological findings. An open biopsy was performed which revealed a metastatic lesion with similar characteristics to the initial endometrial cancer. The lesion had morphological characteristics of dedifferentiated adenocarcinoma, with squamous and small cell differentiation, while in limited positions sarcomatoid features were observed.

**Figure 3:**
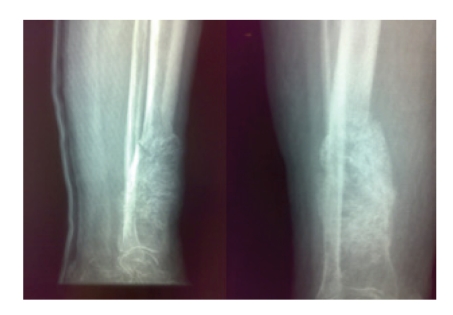
Radiograph of the tibial metastasis showing the increased dimensions and the soft-tissue mass.

The options of palliative radiotherapy or a bellow knee amputation were presented to the patient but no consent was provided by her who initially refused any further treatment. A splint was applied to the tibia and a discharge of the extremity was advised. The patient rereferred to our department 3 months later with an obvious deterioration in the tibial mass with increased dimensions, obvious soft-tissue mass in plain radiographs and skin infiltration (Figure 3). An above-knee amputation was finally performed, while the patient refused to receive chemotherapy. At two years of follow up the patient is alive with no evidence of disease.

## 3. Discussion

Skeletal metastases are the most common variety of malignant bone tumours. They can be solitary or multiple and they could have a lytic, blastic, or mixed appearance mainly based on the origin of the primary tumour. In particular, osteolytic metastases are the most common, representing about 75% of all metastatic lesions [8]. Cancers usually associated with osteolytic metastases are lung cancer, breast cancer, thyroid cancer, kidney cancer, and cancers of the gastrointestinal track such as gastric and colon cancers [8]. A true isolated bone metastasis seldom occurs, with an incidence of only 2% to 3% of all skeletal spread situations [3]. Vertebral metastases are the most common sites for solitary osseous lesions. Solitary bone metastases are usually associated with thyroid gland cancers, melanomas, and renal cancers [8].

Endometrial carcinoma remains one of the most frequent gynecological malignancies occurring predominantly in postmenopausal women. The most common sites of distant metastases from endometrial cancer involve the lymph nodes, the lung, and the liver [4]. The occurrence of bone metastasis secondary to endometrial cancer is very rare. Endometrial metastases to the bone are generally restricted to the vertebrae [4, 9]. The average interval from initial diagnosis to bone metastasis has been reported to be approximately 3 years [10]. Endometrial tumours usually spread to the vertebrae and the pelvic bones via Batson's plexus and systemic vertebral venous plexus [10]. Although the haematogenous spread to the distal extremities could be a possible way for distal metastases of endometrial cancer [5], the exact mechanism remains unknown [11]. The aetiology could be similar to vertebral metastases through the Batson's paravertebral valveless venous plexus that communicates with lower extremities vessels [12]. Another possible theory is that the vascular invasion could start in the lymphatics where tumour cells gain access to the venous return and then to the systemic circulation via arterial outflow [11, 13].

The rarity of the present case report of a tibial metastasis from endometrial cancer consists of two facts: (a) it is a solitary lytic metastasis, distal to the knee, and (b) the primary neoplasm is endometrial adenocarcinoma. Most of endometrial cancers metastatic to the bones were of high stage and often poorly differentiated. The fact that in our case the histological examination of the primary cancer displayed areas of dedifferentiated neoplasm could explain its metastatic propensity. Our literature search identified 5 other reported cases of tibial metastases from endometrial cancer [4, 6, 9, 14, 15]. Finally, another remarkable fact of the present case report is that the patient had a surprising favourable outcome with the aggressive treatment that was chosen, despite the poor prognosis that was expected based on the diagnosis of metastatic dedifferentiated endometrial adenocarcinoma.
